# Effect of Microwave Radiation Power on the Size of Aggregates of ZnO NPs Prepared Using Microwave Solvothermal Synthesis

**DOI:** 10.3390/nano8050343

**Published:** 2018-05-18

**Authors:** Jacek Wojnarowicz, Tadeusz Chudoba, Stanisław Gierlotka, Witold Lojkowski

**Affiliations:** Institute of High Pressure Physics, Polish Academy of Sciences, Sokolowska 29/37, 01-142 Warsaw, Poland; chudoba@unipress.waw.pl (T.C.); xray@unipress.waw.pl (S.G.); w.lojkowski@labnano.pl (W.L.)

**Keywords:** zinc oxide nanoparticles (ZnO NPs), microwave solvothermal synthesis (MSS), microwave reactors, agglomerates and aggregates, ZnO NPs water suspension

## Abstract

This paper reports the possibility of changing the size of zinc oxide nanoparticles (ZnO NPs) aggregates through a change of synthesis parameters. The effect of the changed power of microwave heating on the properties of ZnO NPs obtained by the microwave solvothermal synthesis from zinc acetate dissolved in ethylene glycol was tested for the first time. It was found that the size of ZnO aggregates ranged from 60 to 120 nm depending on the power of microwave radiation used in the synthesis of ZnO NPs. The increase in the microwave radiation power resulted in the reduction of the total synthesis time with simultaneous preservation of the constant size and shape of single ZnO NPs, which were synthesized at a pressure of 4 bar. All the obtained ZnO NPs samples were composed of homogeneous spherical particles that were single crystals with an average size of 27 ± 3 nm with a developed specific surface area of 40 m^2^/g and the skeleton density of 5.18 ± 0.03 g/cm^3^. A model of a mechanism explaining the correlation between the size of aggregates and the power of microwaves was proposed. This method of controlling the average size of ZnO NPs aggregates is presented for the first time and similar investigations are not found in the literature.

## 1. Introduction

The interest in zinc oxide (ZnO) has been dynamically increasing recently [1]. The primary reason is the unique physical and chemical properties of ZnO. It is a II–IV semiconductor characterized by the band gap of 3.37 eV and the exciton binding energy of 60 meV [2]. Selective doping that enables controlling the semiconductor properties of ZnO, such as conductivity or forbidden band, has made it an interesting material for potential application in electronics, spintronics and optoelectronics. The biocompatibility, antibacterial and antifungal action of ZnO are the reasons it enjoys large interest in pharmacy and biomedicine [3,4,5,6,7,8,9]. It is a common ingredient of everyday products, e.g., rubber, pigments, cements, plastics, paints, pharmaceutical products and cosmetics [10,11,12,13].

ZnO nanoparticles (NPs) enjoy constantly growing popularity among scientists worldwide since they offer unique perspectives of application [1,14,15,16,17]. The properties of ZnO NPs can be modified by changing their size, shape, chemical composition and surface area. Industrial synthesis of ZnO NPs with repeatable properties is a rather difficult and troublesome issue. In our earlier publications [18,19,20,21] we discussed the causes of obtaining differing properties of ZnO NPs, which result, among others, from the limitations of the synthesis method, the purity of reagents, and the complexity of chemical reactions. Generally, the primary barrier to obtaining NPs characterized by intended properties is the lack of simultaneous control over average particle size, particle size distribution, shape, phase purity, surface area modification, as well as agglomeration and aggregation.

ZnO NPs are commonly obtained by several methods, among others using the mechanochemical process, precipitation process, sol-gel method, sonochemical technique, hydrothermal and solvothermal method [11]. Nevertheless, microwave-assisted syntheses, e.g., microwave solvothermal synthesis (MSS) [18,19,20,21] and microwave hydrothermal synthesis (MHS) [22,23,24], are some of the most appreciated and dynamically developed methods of obtaining ZnO NPs.

Compared with conventional synthesis methods, the microwave synthesis is faster, purer and more energy-efficient [21,23]. The use of microwaves is an efficient way to supply energy to the reaction vessel, which results in a more homogeneous and rapid heating in comparison with heat transfer through conduction or convection [25]. The impact of microwave heating on the quality of synthesis products is shown in the publication by Rizzuti et al. [26], which discusses microwave synthesis of metastable aragonite with the efficiency reaching as much as 99%, whereas the highest efficiency of aragonite synthesis achieved was merely 61% in the case of conventional heating.

Many forms could be obtained in the microwave synthesis of ZnO nanomaterials (NMs), e.g., spherical particles, rods, flowers, needles, whiskers and layers [11,12,18,24,27,28,29,30,31,32,33,34,35,36,37,38], where the control of ZnO NMs size is possible by changing the synthesis duration, substrate concentration, substrate type, process temperature, solvent type, pH and addition of surfactants [11,18,19,38,39,40,41,42,43]. However, the size control of the forming aggregates is not a very commonly discussed aspect of ZnO NPs synthesis. The definitions of agglomerates and aggregates have been changed over the years both by scientists and the industry, and as a consequence these terms have been often confused [44,45]. Since 2008, when the International Organisation for Standardisation (ISO) introduced a glossary of “Nano-objects” [46,47,48], the terms agglomerate and aggregate are officially defined as follows: agglomerate is a collection of weakly or medium strongly bound particles where the resulting external surface area is similar to the sum of the surface areas of the individual components. The forces holding an agglomerate together are weak forces, for example van der Waals forces or simple physical entanglement; aggregate is a particle comprising strongly bonded or fused particles where the resulting external surface area is significantly smaller than the sum of surface areas of the individual components. The forces holding an aggregate together are strong forces, for example covalent or ionic bonds, or those resulting from sintering or complex physical entanglement, or otherwise combined former primary particles.

ISO has not provided any numerical criteria characterizing the binding forces for the agglomeration and aggregation processes [46,47,48].

The size control of both agglomerates and aggregates of ZnO NPs is particularly important in the aspect of their toxicity [49,50], in the application of ZnO NPs as a filter against UV radiation [51,52], in transparent coatings or films [45,53] and in solar cells, where an optimum homogeneous distribution of controlled nanostructures is required.

Several publications in the relevant literature discuss various ways to control the size of ZnO NPs aggregates, mainly those obtained by the solvothermal method [54,55,56,57,58,59].

Guo et al. [54] obtained ZnO NPs aggregates from a precursor created through mixing Zn(Ac)_2_·2H_2_O-ethanol with NaOH-H_2_O-diethylene glycol (DEG), which was stirred vigorously for 4 h at the temperature of 75 °C. The size of spherical ZnO NPs aggregates ranging from ~90 nm to ~500 nm was controlled by changing the water content in the precursor, while the size of ZnO NPs in aggregates was controlled within the range from 8 nm to 50 nm by the regrowth process at the temperature of 130–180 °C for 4 h.

Another publication describing the synthesis of ZnO NPs aggregates is the paper by Zhang et al. [55], where the impact of the synthesis temperature of ZnO NPs obtained from Zn(Ac)_2_·2H_2_O dissolved in DEG was researched. Regardless of the synthesis temperature of 160–190 °C, ZnO NPs were characterized by the constant size of 15 nm and the specific surface area of ~80 m^2^/g. The equal synthesis duration of 8 h was adopted for all samples [55]. At the temperature of 160 °C, spherical ZnO NPs aggregates with the size ranging from 50 to 350 nm were obtained. At the temperature of 170 °C, aggregates of a deformed spherical shape were obtained. At the temperature of 180 °C, the synthesized sample was composed partly of aggregates and ZnO NPs, while the sample obtained at the temperature of 190 °C was composed of homogeneous ZnO NPs.

Shi et al. [56] described the synthesis of ZnO hierarchical aggregates. The size and shape of ZnO NPs aggregates obtained at the temperature of 60 °C for 8 h was controlled by changing the amount of KOH-methanol solution (0.4–1.6 M) dripped into the solution of Zn(Ac)_2_·2H_2_O-methanol (0.01 M). The morphology of ZnO NPs aggregates composed of 5 nm particles changed from large microspheres to small ellipsoids in line with the increase in the dripped amount of KOH.

Lu et al. [57] described the synthesis of ZnO NPs aggregates in the aqueous solution of Zn(NO_3_)·6H_2_O with an addition of triethanolamine (TEA, (OHC_2_H_4_)_3_N) at the temperature of 200 °C for 3 h. The aggregates were built of ~50 nm ZnO NPs. The size of ZnO NPs aggregates was controlled within the range of 100 nm–1.1 µm through a change of TEA concentration in the precursor (from 5 wt. % to 55 wt. %).

Šarić et al. [58] obtained ZnO NPs aggregates from zinc acetylacetonate monohydrate (Zn(C_5_H_7_O_2_)_2_·H_2_O dissolved in various organic solvents (ethanol and octanol) at the temperature of 170 °C. The aggregates were composed of ~20 nm ZnO NPs. The size of ZnO NPs aggregates was controlled within the range of 0.5 µm–3.5 µm through the change of the molar ratio of TEA to Zn(C_5_H_7_O_2_)_2_·H_2_O; the solvent type and the synthesis duration (4 h, 24 h, 72 h).

Saito et al. [59], synthesized micrometer-sized ZnO spherical aggregates solvothermally from the solutions of Zn(Ac)_2_ dissolved in ethylene glycol (EG) with an addition of hexamethylenetramine (HMT) and water. The size of ZnO NPs in the aggregates was controlled within the range from 5 nm to 30 nm through the change of the synthesis duration (3–12 h) and the temperature (95–120 °C). The size of the obtained ZnO NPs aggregates ranged from 1 to 5 µm.

It is a quite difficult task to obtain ZnO NPs that are completely devoid of agglomerates or aggregates [59]. Although the product obtained during the synthesis in wet methods [60] is characterized by a low degree of agglomeration/aggregation, most often uncontrolled agglomeration and aggregation processes occur at the subsequent stages of rinsing and drying.

A way to reverse the effects of the agglomeration process is the process of deagglomeration of NPs, which consists of breaking the “bonds” between single crystals of nanoparticles in the agglomerates. Interparticle adhesion forces, which make particles connect and stay together, thus forming agglomerates or aggregates, can be reduced by creating a repulsive electrostatic interaction or preventing the contact of particle surfaces between one another through a modification with a polymer layer [61].

Milling, high-shear mixing and ultrasonification are common mechanical processes used for breaking agglomerates down, in particular in suspensions. These processes result both in decomposition of agglomerates and re-agglomeration of particles and the existing agglomerates/aggregates, which considerably broadens the particle size distribution, so it is essential to select optimum deagglomeration parameters individually depending on the type of process and the type of NPs. All the enumerated methods are effective when breaking down agglomerates, i.e., particles which are bonded together by “physical interparticle bonds”, e.g., van der Waals forces, hydrogen bonds, electrostatic interactions. However, in the case of aggregates, i.e., particles which are “bonded together by strong interparticle bonds [52,62]”, all the listed methods are rather ineffective even when very large amounts of energy are supplied. Most research groups do not describe the manner of counteracting the formation of NPs agglomerates and aggregates in their papers, which can be a deliberate action in order not to reveal their technology of powder preparation process (know how) or the presence of agglomerates and aggregates is regarded as irrelevant for the application of NPs.

In our previous publications [18,19,20] we described the manner of controlling the size of ZnO NPs obtained in MSS from the precursor, which was zinc acetate dissolved in ethylene glycol. We found that the size of ZnO NPs obtained in MSS can be precisely controlled within the range from circa 15 nm to 120 nm by changing the water content in the precursor [18]. The mechanism of ZnO NPs size control by changing the water content in the precursor was explained by us in the publication [19].

The aim of this paper was to indicate the possibility to control the size of ZnO NPs aggregates obtained in the microwave solvothermal synthesis by changing the microwave radiation power. Our test results reveal a new method of size control of ZnO NPs aggregates obtained in MSS by using an innovative generation stop-flow microwave reactor [21,22,23,63,64].

## 2. Materials and Methods 

### 2.1. Substrates

The reagents were used in the “as received” condition: zinc acetate dihydrate ((CH_3_COO)_2_Zn∙2H_2_O, (Ac)_2_Zn∙2H_2_O, analytically pure); ethylene glycol (EG, C_2_H_4_(OH)_2_, pure). All reagents were purchased from Chempur (Piekary Śląskie, Poland). Only deionized water (specific conductance <0.1 μS/cm, HLP 20UV, Hydrolab, Straszyn, Poland) was used for the synthesis and preparation process of ZnO samples.

### 2.2. Synthesis of ZnO NPs

ZnO NPs were obtained in accordance with the procedure described in our previous publications [12,18,19]. The solution (900 mL) of zinc acetate dihydrate (0.3037 mol/dm^3^) dissolved in ethylene glycol was prepared using a hot-plate magnetic stirrer (70 °C, 450 rpm, SLR, SI Analytics GmbH, Mainz, Germany). Upon the complete zinc acetate dissolution, the solution was immediately poured into a bottle (1000 mL, polypropylene), which was then sealed. The water (H_2_O) content test in the precursor was carried out once the solution temperature reached room temperature (RT). A properly calculated amount of H_2_O was added to the precursor such that the content was 1.5 wt. %, which was verified.

The synthesis reaction was carried out in a stop-flow microwave reactor, MSS2 model (3 kW, 2.45 GHz, IHPP PAS (Warsaw), ITeE-PIB (Radom), ERTEC (Wroclaw), Poland) [63]. The synthesis parameters are provided in Table 1.

The reactions of ZnO NPs synthesis in ethylene glycol are described by the following general Equation (1):

(1) $${\left({\mathrm{CH}}_{3}\mathrm{COO}\right)}_{2}\mathrm{Zn}+2C_{2}H_{4}{\left(\mathrm{OH}\right)}_{2}\overset{C_{2}H_{4}{\left(\mathrm{OH}\right)}_{2},\;H_{2}O,\;.T,\;.P\;}{\to }\mathrm{ZnO}+H_{2}O+2{\mathrm{CH}}_{3}{\mathrm{COOC}}_{2}H_{4}\mathrm{OH}$$

After the synthesis, the obtained suspensions were sedimented and the supernatants were decanted. The sediments were rinsed three times with water and centrifuged (MPW-350, MPW Med Instruments, Warsaw, Poland). As a result of vigorous stirring of moist powders with water in 100 mL containers, water suspensions of ZnO NPs were obtained from these powders, and subsequently their concentration was determined. Half of the volume of ZnO NPs suspensions was frozen in liquid nitrogen and dried in a freeze dryer (Lyovac GT-2, SRK Systemtechnik GmbH, Riedstadt, Germany).

### 2.3. Water Content Analysis

The content of water in the samples was analyzed in the Cou-Lo AquaMAX KF automatic titrator (GR Scientific, Bedford, UK), which works in line with Karl Fischer’s principles [18,19]. The following reagents were used in the titrator: Aquagent^®^Coulometric OIL anolyte (Scharlau, Barcelona, Spain) and Aquagent^®^Coulometric CG catholyte (Scharlau, Barcelona, Spain). The measurement of weight of liquid samples placed in glass syringes was carried out using the analytical scales (WAA 100/C/1, RADWAG, Radom, Poland).

### 2.4. Morphology Characteristics

The morphology characteristics of the samples were observed in compliance with an internal laboratory measurement procedure (P5.10, edition 6 of 26.08.2015) using the scanning electron microscopy (SEM) (ULTRA PLUS, ZEISS, Oberkochen, Germany). Before inserting the samples into the microscope, the samples were coated with a thin conductive (carbon) layer.

### 2.5. X-ray Powder Diffraction

Diffraction patterns of the X-ray powder diffraction (XRD) were gathered at the RT within the range of 2 theta angle from 10° to 100° with the step of 0.02°, using the X-ray powder diffractometer (CuKα1) (X’Pert PRO, Panalytical, Almelo, The Netherlands). The full width at half maximum (FWHM) was determined by the Pearson VII function implemented in Fityk software, version 0.9.8. Based on the diffraction patterns, the size of crystallites was determined in the direction of the crystallographic axes a and c using Scherrer’s formula [12,18].

### 2.6. Crystallite Size Distribution

For the purpose of determining the crystallite diameter and crystallite size distribution, the FW15/45M method was used [65], which was implemented in Nanopowder XRD Processor Demo, a web application. It should be emphasized that the Nanopowder XRD Processor Demo application employs equations that are dedicated to spherical crystallites. Diffraction files were uploaded directly via http://www.science24.com/xrd website [66]. The data for crystallite size distribution charts were exported from the server on http://science24.com/fw145m/ website [67].

### 2.7. Measurement of Density and Specific Surface Area

Skeleton density, also known as pycnometric density, was measured in compliance with ISO 12154:2014 procedure in the AccuPyc II 1340 helium pycnometer (FoamPyc V1.06, Micromeritics^®^, Norcross, GA, USA) at the temperature of 25 ± 2 °C. The specific surface area (SSA) was measured in compliance with ISO 9277:2010 procedure in the Gemini 2360 surface analyzer (V 2.01, Micromeritics^®^, Norcross, GA, USA) using the Brunauer-Emmett-Teller (BET) method and the nitrogen adsorption-desorption isotherm. The standard deviation of the specific surface area results was calculated using MicroActive software V4.03 (Interactive Data Analysis Software, Micromeritics^®^, Norcross, GA, USA). Both before the density and specific surface area tests, the samples were degassed in the VacPrep 061 degassing station (Micromeritics^®^, Norcross, GA, USA) for 2 h (0.05 mbar, 150 °C). The average particle size (diameter) was calculated based on the specific surface area and density results. The description and limitations of the method as well as the equation employed for the average particle size calculations are included in our earlier publications [12,18,19,20].

### 2.8. Measurement of Average ZnO NPs Size in Water Suspension

Before the dynamic light scattering (DLS) measurement, samples of three ZnO NPs water suspensions with the volume of 80 mL and concentration of 0.05% were subjected to ultrasonic homogenization using the VCX750 ultrasonic processor by Sonics & Materials Inc. (Newtown, CT, USA), with the following homogenization parameters: duration of 4 min, amplitude of 0.7, cycle of 1, solid sonotrode with the diameter of 19 mm, without suspension temperature stabilization. After reaching the room temperature of 22.9 °C, the homogenized suspensions were vigorously stirred and subsequently poured to DTS0012 square polystyrene measurement cuvettes (disposable sizing cuvette) and subjected to analysis.

The average size of ZnO particles in water suspension was measured with the use of Zetasizer Nano-ZS ZEN 3600 analyzer produced by Malvern Instruments Ltd. (Malvern, UK) using DLS. The measurements were carried out in accordance with ISO 22412:2008 standard with the following parameters: temperature of 23 °C, temperature stabilization time of 120 s, measurement angle of 173° (backscattering), analysis model in auto mode. All tests were carried out in DTS0012 cuvettes, while the software used was Zetasizer 7.11, (Malvern Instruments Ltd., Malvern, UK). The average particle size in water suspension analyzed by the DLS method is called Z-Average Particle Size (${\bar{x}}_{DLS}$). The ${\bar{x}}_{DLS}$ results in the DLS method are calculated by the technique of cumulants (ISO 22412:2008) and refer to the diameter (d) of a sphere. The average size of ZnO NPs provided by the DLS method is the average value of the size of particles, agglomerates and aggregates present in the tested suspension sample.

## 3. Results and Discussion

### 3.1. Morphology

**Figure 1:**
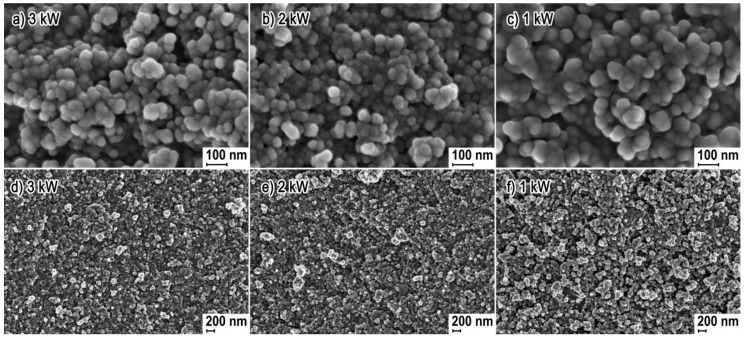
SEM images of products of synthesis of ZnO NPs obtained with the microwave radiation power: (**a**,**d**) ZnO-3 kW; (**b**,**e**) ZnO-2 kW; (**c**,**f**) ZnO-1 kW.

Figure 1 shows representative SEM images of all the obtained samples of ZnO NPs. SEM images no. 1 a, b, c reflect the homogeneous spherical shape of the obtained ZnO NPs with the size ranging from 20 to 50 nm. The ZnO-1 kW sample differed from the remaining ones (image 1c) in the compact structure of particle clusters. NPs in the ZnO-1 kW sample were so compressed together that the carbon layer originating from the use of the sputter coater makes it difficult to determine the size of single NPs of which the agglomerates and aggregates were built, which creates an illusion that NPs in image no. 1c are larger than ZnO-3 kW and ZnO-2 kW. SEM images no. 1 d, e confirm that a homogeneous morphology was achieved for ZnO-3 kW and ZnO-2 kW samples, where a small quantity of structures forming the agglomerates and aggregates can be seen. ZnO-1 kW sample is composed mainly of agglomerates and aggregates, which form a loose powder structure (Figure 1f).

### 3.2. Phase Composition

**Figure 2:**
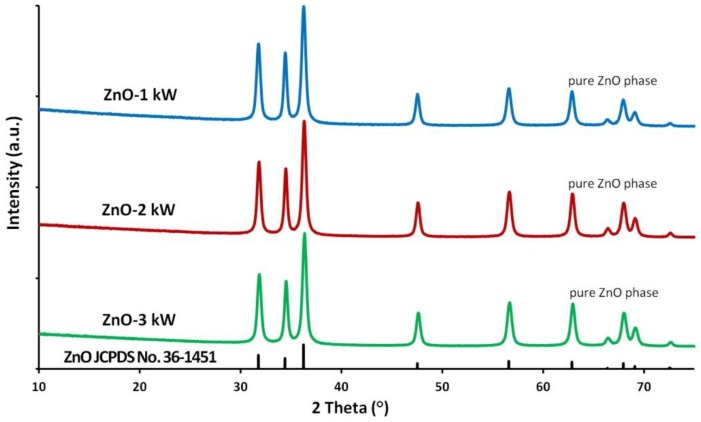
X-ray diffraction patterns of ZnO NPs.

No presence of foreign phases was detected in the obtained ZnO NPs samples. All diffraction peaks shown in Figure 2 come from the hexagonal phase of ZnO, which is reported in PDF card No. 36-1451.

### 3.3. Density, Specific Surface Area and Average Size and Size Distribution of NPs

The results were summarized in Table 2. The specific surface area values of ZnO-3 kW and ZnO-2 kW samples were identical as 40.6 m^2^/g, while the specific surface area of ZnO-1 kW sample was 40.1 m^2^/g. The difference in the specific surface area value between the samples is statistically insignificant and may result solely from measurement error. No impact of the change of microwave radiation power on the skeleton density of the obtained ZnO NPs samples was observed, where all the obtained density results were within standard deviations of these measurements (Table 2).

**Figure 3:**
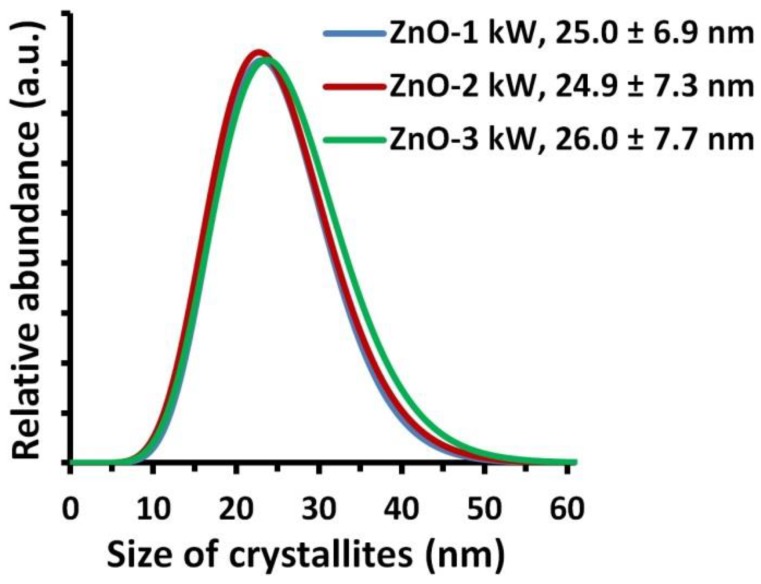
Crystallite size distributions of ZnO NPs obtained using Nanopowder XRD Processor Demo, pre α ver.0.0.8, © Pielaszek Research [66].

The results of average size of the obtained ZnO NPs samples were summarized in Table 2. When comparing the results of average size of crystallites obtained by converting the XRD data using the Scherrer’s formula and Nanopowder XRD Processor Demo, we obtained coinciding results for all samples. The average size of crystallites arising from XRD calculations coincide with the accuracy of 2–4 nm with the results of average particle size obtained based on the results of specific surface area and skeleton density. The differences between the average particle size and the average crystallite size are within the range of standard deviation of the results of the Nanopowder XRD Processor Demo method. The crystallite size distributions of ZnO-2 kW and ZnO-1 kW samples coincide (Figure 3), while the crystallite size distribution for ZnO-3 kW sample is shifted by 1–2 nm towards larger sizes. The obtained differences between crystallite size distributions are statistically insignificant. Based on the obtained results, it can be concluded that:

1. ZnO NPs are built of single crystals.
2. ZnO NPs size is equal to the crystallite size.
3. The change of microwave radiation power did not impact the average size of the obtained ZnO NPs.

### 3.4. Average Size and Size Distribution of ZnO NPs in Water Suspensions

**Figure 4:**
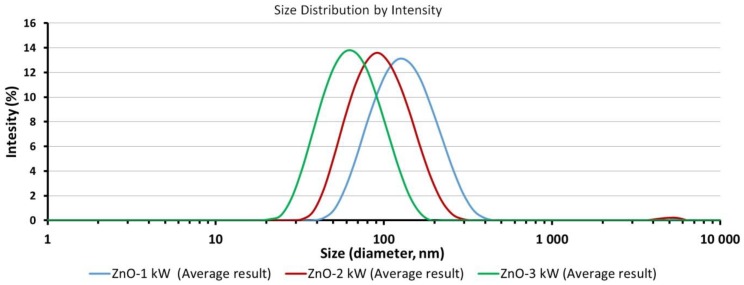
Size distributions of ZnO particles/aggregates obtained by DLS method.

The test results of the average size of ZnO particles/aggregates in water suspensions after ultrasonic homogenization are provided in Table 3. The DLS analysis revealed the following ${\bar{x}}_{DLS}$ results: 59 nm for ZnO-3 kW suspension, 89 nm for ZnO-2 kW suspension and 120 nm for ZnO-1 kW suspension. Only ZnO-3 kW suspension was characterized by a monomodal size distribution (Table 3, Figure 4).

Bimodal distributions were obtained for the remaining two suspensions. Figure 4 for ZnO-2 kW and ZnO-1 kW samples show additional peaks with the intensity below 1% within the range of circa 4700–5000 nm, which are an undesirable effect of the use of ultrasonic homogenization. The polydispersity index values are similar, and objects sized circa 4700–5000 nm do not affect the overall evaluation of the discussed correlations. When comparing the particle size distributions of ZnO NPs suspensions presented in Figure 4, it can be noticed that all distributions presented on the logarithmic scale are similar in terms of width, while peak maximums are shifted in relation to each other by a multiple of the average size of a single ZnO (~30 nm) particle. The DLS results revealed the possibility to control the size of ZnO NPs aggregates by changing the used microwave radiation power with simultaneous preservation of the constant size of ZnO NPs.

For dispersing ZnO NPs in deionized water, we used the high-energy ultrasonic method, which is very effective in breaking agglomerates down. Therefore, we argue that the ${\bar{x}}_{DLS}$ results obtained by us refer mainly to the average size of particles and aggregates which were present in the suspension after the ultrasonic homogenization. We cannot confirm this conclusion with differences in the specific surface area results (Table 2), which would be consistent with the official definition of aggregate (ISO [46,47,48]), since the obtained differences between specific surface areas of individual samples were slight. Similar results were obtained by Zhang et al. [55], who reported obtaining spherical aggregates sized 50–350 nm, whereby all samples obtained by them had almost identical specific surface areas of circa 80 m^2^/g. The lack of significant differences in specific surface area values of samples with different sizes of ZnO NPs aggregates may indicate a negligible impact of the contact of surfaces of single ZnO nanoparticles on the change of the total specific surface area of ZnO NPs aggregates obtained by the solvothermal method.

**Figure 5:**
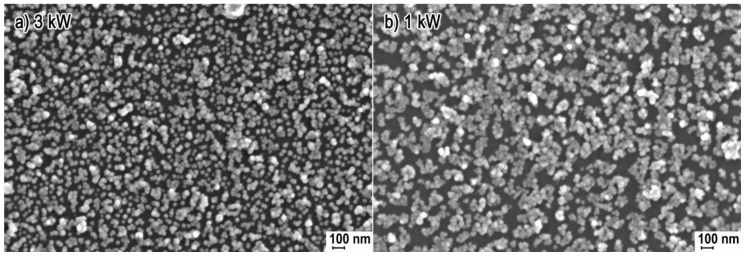
SEM images of ZnO NPs layers obtained on polyurethane film using the ultrasonic method from NPs: (**a**) ZnO-3 kW; (**b**) ZnO-1 kW.

In order to demonstrate the differences in the size of the obtained ZnO NPs aggregates, we coated polyurethane film with ZnO-3 kW and ZnO-1 kW nanoparticles. The applied ultrasonic coating method was described in our previous publication [53]. We used identical coating parameters for two samples of the same polyurethane film: suspension concentration of 0.05%, duration of 15 min, amplitude of 50%. The following SEM images (Figure 5) show a considerable difference in the morphology of the obtained ZnO NPs layers.

SEM image no. 5a presenting a ZnO-3 kW NPs layer shows the prevailing quantity of single ZnO particles and small clusters of ZnO agglomerates/aggregates, which is consistent with the DLS results. SEM image no. 5b, in turn, presents a layer obtained from ZnO-1 kW NPs, where the majority of the layer is composed of compact clusters of agglomerates/aggregates and sporadic quantities of single ZnO NPs. The obtained film layer from ZnO-3 kW NPs is more homogeneous in comparison with the ZnO-1 kW NPs coating. When discussing the obtained layers, it should be pointed out that a powder composed of a greater quantity of and larger aggregates during ultrasonic coating simultaneously forms the major amount of secondary agglomerates/aggregates on the film surface, which can be observed when comparing SEM image no. 5a with no. 5b. The size of secondary agglomerates/aggregates for ZnO-1 kW NPs ranges from 150 to 300 nm, i.e., are 2–3 times greater than the average particle/aggregate size measured using DLS, which was 120 nm.

### 3.5. Dependence of Pressure on Ethylene Glycol Temperature

In order to discuss the impact of synthesis parameters on the change of size of ZnO NPs aggregates, we additionally measured the actual temperature of the microwave heating process in the MSS2 reactor.

**Figure 6:**
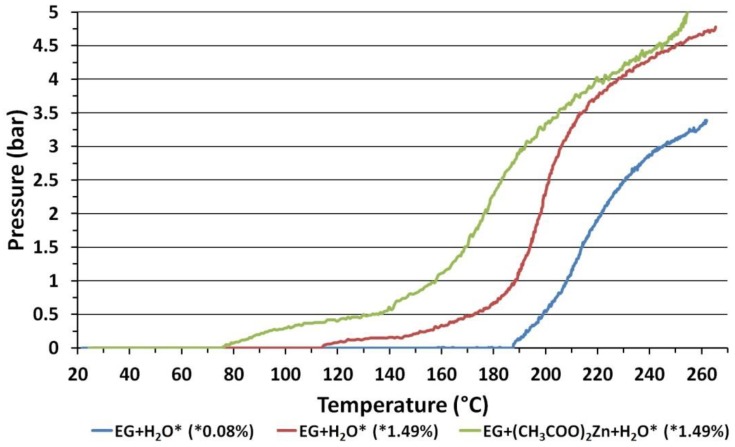
Chart presenting the relation between pressure and temperature for: EG with 0.08 wt. % of H_2_O, EG with 1.49 wt. % of H_2_O and precursor (Ac)_2_Zn·2H_2_O dissolved in EG with 1.49 wt. % of H_2_O. Experimental data obtained in the MSS2 microwave reactor.

The MSS2 reactor enables controlling the process only through measurement of actual pressure [63]. In order to determine the ZnO NPs synthesis temperature in the MSS2 reactor, we constructed a specialized Teflon^®^ batch chamber. The temperature was recorded with the use of a K-type thermocouple (Ø1 mm, T-208p-K-1, Termo-Precyzja, Wroclaw, Poland), which was inserted inside the chamber in a Teflon^®^ jacket. The thermocouple was placed centrically in the bottom wall of the chamber, while the thermocouple end was at a distance of 3 cm from the bottom wall of the chamber. The results of temperature measurements for the heating processes of 270 mL of feedstock with the use of microwave radiation power of 1 kW are presented in Figure 6.

We used pure EG (0.08 wt. % of H_2_O) as a reference sample with a known boiling point of ≈197 °C. Figure 6 indicates that while heating the EG sample the pressure increased for the temperature of ≈187 °C. This proves that the thermocouple placed in the reactor chamber measures the temperature with a delay of several seconds and the temperature measurement is lower by circa 10 °C in relation to the pressure measurement. This is caused by the use of the Teflon^®^ jacket, which provided chamber tightness and at the same time acted as thermal insulation [23]. The differing temperatures of pressure increase while heating individual samples (Figure 6) results from their differing compositions. Taking into account measurement error, the ZnO NPs synthesis temperature in the MSS2 reactor for 4 bar was ~230 °C.

### 3.6. ZnO NPs Synthesis Temperature

The correlation between the size of ZnO NPs aggregates and the microwave radiation power, which we observed, can be explained by the mechanism of solvothermal synthesis of ZnO NPs [19], which must take into account the impact of heating kinetics on the reaction course.

The course of the microwave synthesis is composed of 3 stages (durations):

- First stage is the duration of feedstock pre-heating until synthesis parameters are reached;
- Second stage is the heating duration for constant synthesis parameters;
- Third stage is the duration of reactor cooling down.

**Figure 7:**
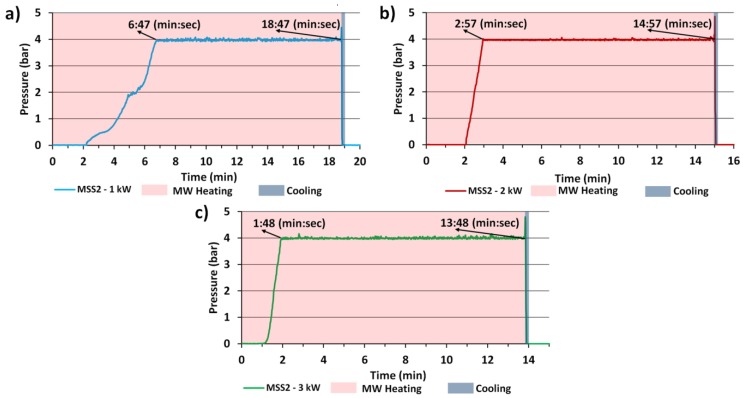
Charts of synthesis parameters for different microwave radiation powers: (**a**) 1 kW; (**b**) 2 kW; (**c**) 3 kW. Source: experimental data from the MSS2 reactor.

Figure 7 present charts of parameters from the course of ZnO NPs syntheses in the MSS2 reactor, in which these syntheses were controlled by pressure. Table 4 summarizes individual durations of synthesis stages. The temperature of circa ~230 °C (Figure 6) was reached for the adopted synthesis pressure of 4 bar. The duration of the first stage, i.e., of reaching the preset parameters, was different in all syntheses, which was consistent with the heating efficiency that depended on the applied power. Figure 7 and Table 4 show a significant impact of the change of microwave power only on the first stage of ZnO NPs synthesis.

The change of the microwave power did not impact the duration of the second stage of the syntheses or the duration of cooling down, which lasted merely a few seconds (Figure 7). The stop-flow design of the MSS2 reaction enabled us to empty the chamber exactly on the moment of finishing the microwave heating, thanks to which the reaction products were rapidly cooled down by more than 140 °C over a few seconds, which immediately stopped the course of any and all chemical reactions. In batch reactors, in turn, e.g., 02-02 model by Ertec Poland, which we used for the MSS of ZnO NPs in our earlier papers [18], the duration of the cooling down alone, after which we could safely empty the reaction chamber, was 20 min. During such a long cooling down, it is possible that the reactions can progress further in an uncontrolled manner, e.g., leading to the formation of ZnO NPs aggregates or to the degradation of the organic solvent.

We did not observe any impact of the total synthesis duration on the size of the obtained ZnO NPs (Table 2), which is consistent with the results from our previous publications [18,19], where we proved that:

1. The size of ZnO NPs in the MSS is the function of H_2_O content in the zinc acetate solution in ethylene glycol.
2. The size of ZnO NPs increases until the unreacted zinc acetate is exhausted.
3. The solvent used by us, ethylene glycol, acts as a stabilizing agent, which eliminates uncontrolled particle growth.

The course of the ZnO NPs synthesis reaction is completely different in a water environment, in turn, which results from the variety of the reagents used and at the same time from the quantity of possible competitive reaction mechanisms. In the hydrothermal synthesis of ZnO NPs e.g., Hasanpoor et al. [27] report creation of ZnO nanostructures (NSs) from aqueous solutions of zinc nitrate-6-hydrate, zinc acetate dehydrate and hydrazine hydrate and ammonia. The increased synthesis duration in their tests resulted in an increase in the size of needle-shaped particles, but after they increased the microwave power the needle-shaped particles completely changed into flower-type ones. Similar results were obtained by Promnopasa et al. [68] who described a mixture of ZnO nanorods and nanoplates obtained from a water suspension of zinc acetate and sodium hydroxide. They also observed an impact of the microwave power increase on the increase in the size of the obtained ZnO NSs and the change of ZnO shape from nanoplates to nanorods. Nevertheless, the change of power (1–3 kW) and of the total synthesis duration (13:48–18:47, min:s) did not impact the size and shape of ZnO NPs in the microwave solvothermal synthesis technology developed by us. The results collected by us demonstrate that certain properties of ZnO NPs are affected to the decisive extent by the precursor composition rather than the parameters of microwave solvothermal synthesis.

The mechanism of MSS of ZnO NPs obtained from (Ac)_2_Zn·2H_2_O dissolved in EG was explained and verified by us [19]. Equations (2)–(4) describe the major stages of the course of the microwave synthesis [19]:

(2) $$5{\left(\mathrm{Ac}\right)}_{2}\mathrm{Zn}+8C_{2}H_{4}{\left(\mathrm{OH}\right)}_{2}+{\mathrm{xH}}_{2}O\;\overset{\mathrm{MSS}\;\left(1^{st}\;\mathrm{stage};\;↑T\right)\;}{\to }{\mathrm{Zn}}_{5}{\left(\mathrm{OH}\right)}_{8}{\left(\mathrm{Ac}\right)}_{2}\cdot {\mathrm{xH}}_{2}O+8{\mathrm{AcC}}_{2}H_{4}\mathrm{OH}$$

(3) $${\mathrm{Zn}}_{5}{\left(\mathrm{OH}\right)}_{8}{\left(\mathrm{Ac}\right)}_{2}\cdot {\mathrm{xH}}_{2}O+2C_{2}H_{4}{\left(\mathrm{OH}\right)}_{2}\;\overset{\mathrm{MSS}\;\left(1^{st}\;\mathrm{stage};\;↑T\right)\;}{\to }\;5{\mathrm{ZnO}}_{NPs}+2{\mathrm{AcC}}_{2}H_{4}\mathrm{OH}+5H_{2}O+{\mathrm{xH}}_{2}O$$

(4) $$\mathrm{ZnO}+{\left(\mathrm{Ac}\right)}_{2}\mathrm{Zn}+2C_{2}H_{4}{\left(\mathrm{OH}\right)}_{2}\overset{\mathrm{MSS}\;\left(2^{nd}\;\mathrm{stage};\;∼230\;{}^{\circ}C,\;.4\mathrm{bar}\right)\;}{\to }\left(1+1\right){\mathrm{ZnO}}_{\left(\mathrm{growth}\right)}+H_{2}O+2{\mathrm{AcC}}_{2}H_{4}\mathrm{OH}$$

**Figure 8:**
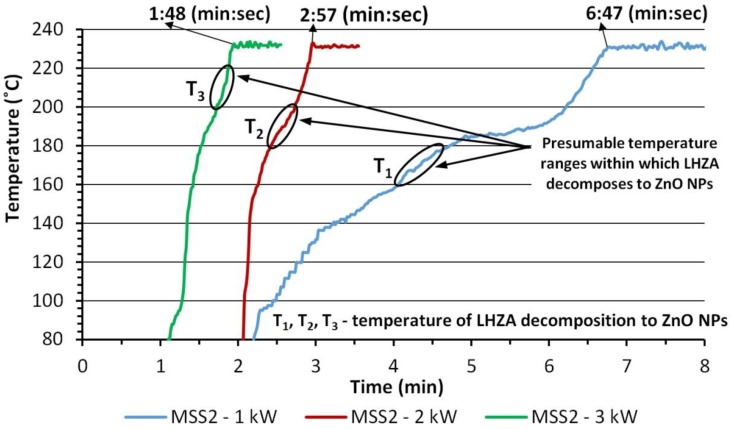
Summary of temperature growth profiles for ZnO NPs synthesis in the MSS2 reactor. Source: own data calculated based on the T = f(P) correlation, where: T—temperature, P—pressure in the reactor, f(P)—sixth-order polynomial function.

We proved that Zn_5_(OH)_8_(Ac)_2_·xH_2_O (lamellar hydroxy zinc acetate, LHZA) is the intermediate (2) of the microwave solvothermal synthesis of ZnO NPs, which rapidly decomposes to spherical 17 ± 2 nm ZnO NPs (3) on reaching the esterification equilibrium constant in the reaction suspension under the influence of temperature. The greater the microwave radiation power was used, the shorter the duration of the first stage of synthesis was, and at the same time the greater the heating speed was (Table 4, Figure 8). 

**Figure 9:**
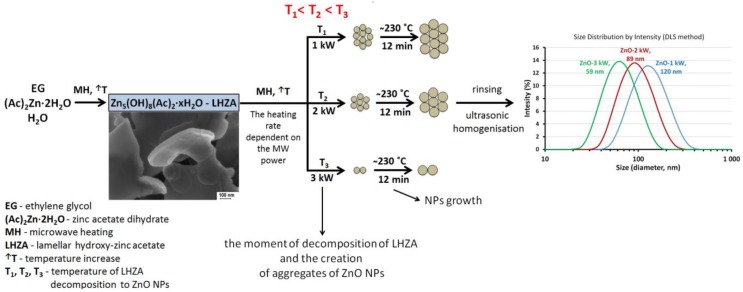
Indicative illustration of formation of ZnO NPs aggregates during the MSS.

We argue that the decomposition of LHZA to ZnO NPs in the MSS2 reactor occurred at the first stage of synthesis, during which the pressure rose to the preset value of 4 bar, i.e., ~230 °C (Figure 8). The microwave heating speed contributes to the change of the time of reaching the esterification equilibrium constant, i.e., decomposition of LHZA to ZnO NPs. Therefore, we presume that the change of heating speed changes the temperature of LHZA decomposition, namely the greater the effective power of microwave heating was, the higher the temperature of LHZA decomposition was reached, which is shown in Figure 8 and Figure 9. The higher the temperature of LHZA decomposition to ZnO is, the more rapidly the process occurs, thanks to which particles are e.g., torn apart more effectively, which would explain the effect of microwave radiation power on the size of the obtained ZnO NPs aggregates (60 nm, 90 nm and 120 nm). However, the mechanism of formation of homogeneous ZnO NPs through LHZA decomposition is unknown to us. This requires further research.

Hypotheses concerning the mechanism of formation of ZnO NPs aggregates can be based on our tests and on the results from the publication by Zhang et al. [55], which describes the impact of synthesis temperature on the structure of ZnO NPs aggregates formed in diethylene glycol. The aforementioned authors obtained spherical aggregates sized 50–350 nm at the temperature of 160 °C, while at the synthesis temperature higher by 30 °C such ZnO NPs aggregates that would be noticeable in SEM images did not form. The size of ZnO NPs was estimated by them at circa 15 nm for all samples despite differences in the synthesis temperature and the obtained morphology.

We presume that homogeneous ZnO NPs formed as a result of LHZA decomposition can be connected with each other via Zn_5_(OH)_8_(Ac)_2_·xH_2_O bridges or ester bridges that form as a result of the reaction of polyols with carboxyl groups on the surface of particles. The durability of ester bridges depends on the synthesis temperature, while Zn_5_(OH)_8_(Ac)_2_·xH_2_O bridges create stable connections between ZnO NPs surfaces under the influence of temperature. In order to determine the type of interparticle bonds or interactions that form ZnO NPs aggregates, further tests must be carried out. Certainly, the final size of ZnO NPs aggregates in the microwave synthesis will be affected by the temperature of ZnO NPs synthesis [55]. However, our test results are the first to report the impact of temperature of LHZA (the intermediate) decomposition on the size of the obtained ZnO NPs aggregates with the constant temperature of the solvothermal synthesis preserved.

## 4. Conclusions

This method of controlling the average size of ZnO NPs aggregates is presented for the first time and similar investigations are not found in the literature.

A method of controlling the average size of ZnO NPs aggregates obtained in the microwave solvothermal synthesis was achieved, which consists of changing the precursor heating speed by changing the microwave radiation power.

The phenomenon of ZnO NPs aggregation is related to a certain temperature range, in which the intermediate (LHZA) decomposes to ZnO NPs and only thanks to that can the change of the heating speed enable controlling the size of the obtained ZnO NPs aggregates with the following average sizes: 60, 90 and 120 nm.

The increase in the microwave power caused a significant change in the heating speed and at the same time a decrease of the time of reaching the pressure of 4 bar in the MSS2 reactor.

The change in the microwave power and the change in the total synthesis duration does not affect the average size of ZnO crystallites obtained from the precursor with a constant composition (EG, (Ac)_2_Zn·2H_2_O, H_2_O) in the case of microwave solvothermal synthesis.

Our paper shows the unique potential of application of stop-flow microwave reactors for controlling the size of ZnO NPs aggregates, which are composed of homogeneous ZnO NPs with a constant size.

Without accurate characterization, which deliberately does not take into account the measurements of size of NPs ZnO aggregates, it is possible to unwittingly obtain different sizes of NPs aggregates with the constant average size of single ZnO NPs preserved, which cannot be repeated. This may contribute to obtaining batches of ZnO NPs characterized by completely different properties, e.g., toxicity.

## Figures and Tables

**Table 1:** Parameters of solvothermal synthesis of ZnO NPs.

Sample Name	Synthesis Duration (min)	Synthesis Pressure (bar)	Microwave Power (kW)	Feedstock Volume (mL)	Power Per 1 mL of Feedstock (W/mL)
ZnO-1 kW	12	4	1	270	3.7
ZnO-2 kW	12	4	2	270	7.4
ZnO-3 kW	12	4	3	270	11.1

**Table 2:** Properties of products of ZnO NPs synthesis.

Sample Name	Specific Surface Area, a_s_ ± σ (m^2^/g)	Skeleton Density, ρ_s_ ± σ (g/cm^3^)	Average Particle Size from SSA, d (nm)	Average Crystallite Size, Scherrer’s Formula, d_a_, d_c_ (nm)	Average Crystallite Size, Nanopowder XRD Processor Demo, d ± σ (nm)
ZnO-1 kW	40.1 ± 0.1	5.16 ± 0.06	29.0	21a; 29c	25.0 ± 6.9
ZnO-2 kW	40.6 ± 0.1	5.19 ± 0.03	28.5	24a; 28c	24.9 ± 7.3
ZnO-3 kW	40.6 ± 0.1	5.18 ± 0.03	28.6	25a; 30c	26.0 ± 7.7

**Table 3:** Results of average ZnO particle size based on DLS analysis.

Suspension Name	ZnO NPs Concentration in H_2_O (%)	Average Diameter, ${\bar{x}}_{\mathrm{DLS}}$(nm)	Polydispersity Index, PI	Distribution Type	Average Size for Peak–Peak Intensity (nm-%)
ZnO-1 kW	0.05	120	0.149	Bimodal	141-99.8; 4714-0.2
ZnO-2 kW	0.05	89	0.156	Bimodal	102-99.4; 4909-0.6
ZnO-3 kW	0.05	59	0.141	Monomodal	68-100

**Table 4:** Course of ZnO NPs syntheses in the MSS2 reactor.

Sample Name	1st Stage—Pre-Heating Duration, 0→4 bar, (min:s)	2nd Stage—Heating Duration, 4 bar, ~230 °C, (min:s)	3rd Stage—Cooling down Duration, ~230 °C→~90 °C	Total Synthesis Duration (min:s)
ZnO-1 kW	6:47	12:00	a few seconds	~18:47
ZnO-2 kW	2:57	12:00	a few seconds	~14:57
ZnO-3 kW	1:48	12:00	a few seconds	~13:48
